# Improving participation of culturally and linguistically diverse participants in clinical trials: an expert consultation

**DOI:** 10.1186/s13063-025-08803-z

**Published:** 2025-03-25

**Authors:** Eliza Watson, Hannah Gulline, Stephen M. Jane, Anne Woollett, Darshini Ayton

**Affiliations:** 1 Health and Social Care Unit, School of Public Health and Preventive Medicine, Monash University, Melbourne, Australia; 2 School of Translational Medicine, Monash University Alfred Hospital Precinct, Melbourne, Australia; 3 Trial Hub, Alfred Health, Melbourne, Australia

**Keywords:** Clinical trials as topic, Diversity, Inclusion, Culturally and linguistically diverse, Ethnic and racial minorities, Cultural diversity, Healthcare disparities

## Abstract

**Background:**

Diversity and inclusivity have become increasingly important in the design and implementation of clinical trials. However, those from culturally and linguistically diverse (CALD) backgrounds are still underrepresented in the research landscape. Failing to include diverse participants can result in treatments and interventions that are not accessible to all who need them. Researchers in Australia and internationally are innovating new ways to address the barriers to increased participation of people from CALD backgrounds in clinical trials.

**Consultation and review:**

We conducted a brief review, augmented by consultation with experts who have engaged CALD communities in research and who hold positions in diversity and inclusivity improvement. Through this, we identified three pillars that must be considered in all areas of design and implementation of trials and research projects: co-design the process of engagement, build trust, invest the time. We also identified seven areas for action where organisations and research teams can focus their activities to improve inclusion and diversity: toolkits and study design, building trust with CALD communities, education and awareness, staff training and communication, language and consent, logistics, resources: funding and time. Importantly, accurate collection of data related to CALD status is also needed to improve inclusivity.

**Conclusion:**

Experts provided valuable insights from their own experiences of the most effective methods for improving the inclusion of CALD communities in clinical trials. Early and thorough planning, building long-term, mutually beneficial relationships with CALD communities and top-down changes to funding are all necessary elements to creating effective, sustainable improvements to the diversity of clinical trials.

## Background

The ethnic diversity and inclusivity of trials have become increasingly important in order to address historical racial inequities and systemic failures and improve understanding of treatments and conditions in diverse populations [1, 2]. Studies that fail to involve people from ethnic minorities are likely to widen health inequalities, by developing interventions that fail to be inclusive or accessible [3, 4]. Despite this, those from culturally and linguistically diverse (CALD) populations are typically underrepresented in clinical trials [5–8]. In Australia, improving the inclusivity of clinical trials is particularly vital given the diverse population, with more than a quarter of people born overseas and almost 23% of people speaking a language other than English at home [9]. Leaders in health and research industries have recognised the need to improve the diversity of clinical trial participants, however, there are many barriers to be addressed to improve participation [1]. Barriers to participation of those from CALD populations may be due to individual, psychological and motivational processes, or external, physical, social and organisational processes [10]. Understanding these barriers and how they have been or can be addressed will lead to improved clinical trial procedures that are more inclusive, and in turn, more comprehensive treatments and interventions. In Australia and internationally, researchers are innovating new ways to address the barriers to increased participation of people from CALD backgrounds. This commentary aims to identify these innovations, discuss barriers and provide practical solutions for future research.

## Understanding the landscape of current practice

We conducted a brief review of recent grey and academic literature to understand common barriers to improved participation of CALD communities, and activities and interventions aimed at addressing these. In addition to this brief review, we consulted experts in health, research and diversity and inclusion who have engaged CALD communities in research studies and trials to understand their experiences and first-hand accounts of working with CALD communities. We spoke to 12 experts (six from Australia, four from the U.K. and two from the U.S.) ranging from senior lecturers, directors and chairs of research, equity or diversity, and leads of trials and centres focused on inclusion. Experts themselves came from CALD and non-CALD backgrounds. These experts approved the inclusion of their discussion and were given the opportunity to review their inclusion in this commentary. Comments from the consulted experts have been incorporated into the text and key quotes highlighted where relevant.

## Defining and reporting CALD status

The way in which CALD is defined, recorded and reported is important for enabling appropriate inclusion in trials and can assist in providing individualised medical care. However, variables that define CALD status are often poorly recorded or inadequate for identifying people from CALD backgrounds [11]. Experts commented on poor documentation of CALD status in medical records leading to difficulties in understanding which CALD groups were most impacted by diseases or medical conditions, and in turn, identifying participants from relevant CALD communities to participate in relevant trials.

In 1999, the Australian Bureau of Statistics defined a minimum core set of four variables for defining CALD status to be collected in government, academic and private sector organisations, alongside several non-core variables. These four variables are the country of birth of the person, main language other than English spoken at home, proficiency in spoken English and Indigenous status [12]. However, these four variables are frequently not collected in full, with the country of birth relied upon as the sole indicator of CALD status [4, 11, 12]. Country of birth can be problematic when used as a single measure as it does not take into account ethnocultural or linguistic information [11]. Furthermore, minimising CALD status to any single variable reduces the pool of people who may otherwise be classified as CALD, and increases the risk of not capturing many who may benefit from access to trials or materials designed for those from CALD communities [11]. Pham et al. [4] recommend that researchers capture as many CALD components as possible to more broadly represent CALD communities, making sure to include country of birth, language spoken at home and indigenous status. Allowing participants to self-describe their ethnicity and involving community members in developing the wording of questions about ethnicity can also improve data collection [13]. Importantly, variables of CALD status must be accurately collected both in the medical setting, to aid in identification for trials and requirement for translated materials or interpreters, and within the trial to improve understanding and generalisability of results.

## Pillars to improved participation of CALD communities in research

We identified three key pillars to inclusion based on our review and expert consultation. These pillars are elements that should be considered in the design and development of all interventions and study design decisions.

Key pillars to improved participation of CALD communities:

- Co-design the processes of engagement
- Build trust
- Invest the time

### Co-design

Co-design should be considered and employed across all areas for action for improving participation of CALD communities. As one expert noted, “each community deserves a tailored approach” and it is important to accommodate for cultural and spiritual nuances and to address the specific needs of different CALD communities [3]. Involvement of members of CALD communities in trial design will ensure that their needs, perspectives and priorities are understood. A majority of the experts consulted discussed working with consumers and members of relevant CALD communities to develop materials, resources or trial design to improve inclusion [14].

**Fig 1:**
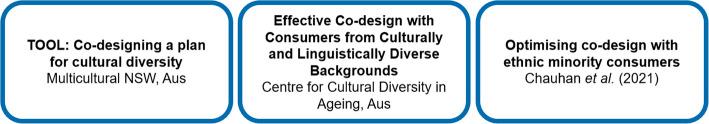
Resources for improving co-design in research with CALD communities

Co-design comes in many different forms and can be employed in different ways depending on the desired outcome [15]. For more meaningful engagement, researchers should aim to collaborate with and empower CALD community participants and leaders [15]. Several resources exist for understanding how best to involve CALD communities in co-design, some key examples are presented in Fig. 1 [16–18].

Co-design can also be employed outside of individual research projects at an organisational and sponsor level, including representation of people from CALD backgrounds in consumer research networks, human research ethics committees and funding bodies to ensure a systemic approach to diverse representation which will help to create change at all levels and prevent perpetuation of existing inequalities [14, 19, 20].

### Build trust

Building trust within CALD communities is vital to improving participation in clinical trials and research and was one of the most commonly discussed themes by the experts we consulted. Fear of participation, a lack of trust of trial procedures, doctors, drugs and the medical industry, as well as a lack of understanding about the value of research within CALD communities, are factors that reduce willingness to participate in trials [3, 21, 22]. Additionally, some community members may have distrust of medical research due to the historical mistreatment of their communities [22, 23]. It is important that trust is built by working with local organisations to develop relationships with target communities [3, 19–22, 24].

### Invest the time

The additional time required to design and implement actions for improved participation of CALD communities in research is a key consideration in creating inclusive research environments. From the time needed to approach and connect with communities, to training staff, developing translated materials and spending additional time with CALD participants to explain trial concepts and build rapport, time comes into all facets of inclusive research. When asked what the biggest barriers to improving CALD participation in research were, a vast majority of those consulted stated that it is time. Thoughtful inclusion of CALD communities requires an authentic connection that cannot be rushed. A more thorough understanding of the activities required to boost diverse participation allows research teams to more accurately budget for their time, while sponsors and funding bodies must also be responsive to, and encouraging of, these requirements.

## Areas of action for improved participation of CALD communities in research

We identified seven areas of action based on our review and expert consultation. These are areas where organisations and research teams can focus their activities to improve participation.

Areas of action for improved participation of CALD communities:

- Toolkits and study design
- Building trust with CALD communities
- Education and awareness
- Staff training and communication
- Language and consent
- Logistics
- Resources: funding and time

### Toolkits and study design

**Fig 2:**
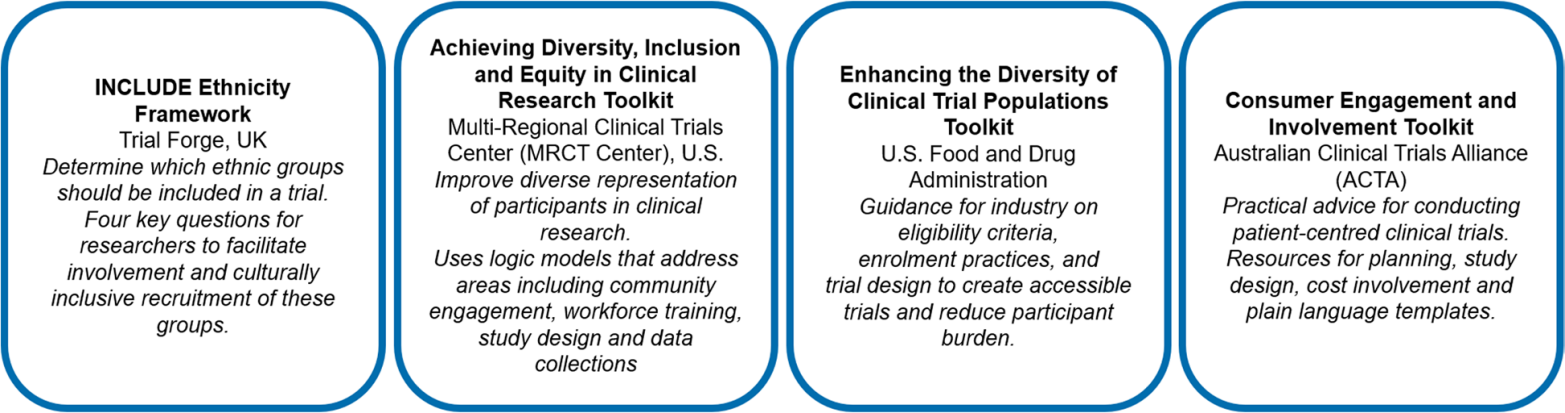
Title, country of origin and purpose of key toolkits and guidance documents for designing inclusive studies

Actions to improve the participation of CALD communities must begin as early as possible in the design of a trial. Four toolkits intended to assist with inclusive and diverse study design, appropriate eligibility criteria and conducting patient-centred trials are presented in Fig. 2. [20, 25–28]

Researchers have reported that the INCLUDE Framework was useful and thought-provoking, however, others found it difficult to put the results of the framework into practice or to find time to complete it [29]. The Multi-regional Clinical Trials Center (MRCT Center) toolkit has not been formally evaluated, but it has received positive feedback from sponsors, and industry and academic medical institutions that it was instrumental in helping them develop their diversity plans [27]. The Australian Clinical Trials Alliance (ACTA) toolkit does not specifically focus on diversity and inclusivity, but it provides resources for inclusivity that can be used to assist in planning and study design [28].

To create a truly inclusive trial requires planning to integrate actions for improving diversity and inclusivity throughout the trial period.

### Building trust with CALD communities

It is vital to create sustainable, authentic, respectful relationships with CALD communities to develop trust and work effectively. Experts discussed connecting with communities in ways that are specific to them and recognise their cultural backgrounds and their historical experiences of research and the medical setting. Cultural brokers, including community liaisons, patient advocates, interpreters and staff with a background representative of the target community can build relationships effectively with communities.

If you want to genuinely make a difference, you’ve really got to immerse yourself in the community and get in as early as possible

the methods of recruitment are going to need to be tailored for those different populations and I think having consumer or community representatives plus people from diverse cultural groups on the team makes it more likely that that tailoring can occur effectively.

Research teams must work with CALD communities to understand their priorities, include them in the research process, and maintain engagement following the conclusion of trials through dissemination of findings [2, 27]. Research teams must also consider working together to streamline their efforts and reduce the burden on communities.

### Education and awareness

CALD communities may be unwilling to participate due to a lack of knowledge of trials and research procedures. Educating communities about research and clinical trials helps to break down misconceptions, provides transparency about the research process and how data is used, and improves understanding of the benefits of participating [23]. Several examples of education for CALD communities are available from Australia, the UK and the USA [30–37]. These examples use different formats including videos in English and in relevant languages for the local communities, and websites with resources and information in English, covering topics such as what clinical trials are, the benefits of participating, how consent works, where data is stored and personal stories from participants. Further evaluation is needed for some of these initiatives in future to better understand their impact on improving uptake of clinical trials in CALD communities, however, they are valuable resources for researchers to understand how to communicate appropriately about research with CALD participants.

### Staff training and communication

Researchers and those involved in trials must be mindful that their behaviour creates an inclusive, respectful environment. Researchers must reinforce the personal health and safety of participants, be open about risks and challenges, and explain the individual, familial and community benefits of participating to the participant [24, 38]. It is important that appropriate communication is maintained throughout the trial process, with staff demonstrating appreciation to participants and calling or emailing to check in frequently [21, 24, 38]. Training for staff may improve cultural competency, understanding of health inequities, dispel incorrect cultural stereotypes, and provide practical communication skills and ways to work more effectively with interpreters and translators [1, 3, 19, 21, 39]. One expert noted the need to develop a mindset that everyone who can potentially benefit from a study has the right to take part.

I think it’s a lot more than cultural humility and cultural sensitivity. I think it’s … developing that mindset that you know everyone who can potentially benefit from this study has their right to take part in the study… we could all do with some training in terms of raising our awareness of how we can do [better]

The Race Equality Framework [40] and the MRCT toolkit [27] (described above) may be useful to assist organisations in understanding gaps in their practice and necessary workforce development to address these.

### Language and consent

Language is one of the most prominent barriers to trial participation, and fluency in English is often a key inclusion criteria [5, 14]. Issues can arise due to limited ability to speak or read English, low literacy levels in English or in a participant’s first language [21]. Trial materials may need to be produced in plain English and in languages relevant to the communities being approached, and tailored to cultural preferences and literacy levels [24, 38]. The consent process can be a point of difficulty for participation, particularly if English proficiency is low, with consent forms often being too long and at an inappropriate readability level, with complicated medical jargon that is overwhelming for participants [41, 42]. Simplification of consent processes, the language used, and alternative means for providing consent such as videos may assist CALD participants and could also benefit English speakers [19, 38, 41, 43]. Experts discussed working with members of CALD communities to co-design simplified video consent and noted the need to discuss simplification with their local ethics committee and legal representatives to ensure the consent process met requirements. Translation is a complex, multi-stage and often time-consuming process. Straight translations from an English text can result in inaccurate or culturally inappropriate language. One expert described a situation where the language of target participants did not have an appropriate word for trial, leading the translators to instead use the word for experiment, which evoked negative connotations and discouraged people from participating. It is vital that translated materials are co-designed with members of the target CALD community and experienced translators.

[English documents] sometimes do not translate well into other languages, and so what’s happening is that the key messages can be literally lost in translation

The process of gaining consent from potential CALD participants can also come with difficulties, including complex socio-cultural factors, limited health literacy, and language barriers [33, 44]. Additional time is often required to explain studies where interpreters are required. However, experts reflected on interpreters at hospitals having a lack of experience in the clinical trial space and recommended training to improve knowledge of trial jargon. Additionally, in the hospital setting, study materials may only be translated into the most commonly spoken languages of the local community, which does not leave room for cultural nuance in the translation where more than one community speak the same language, and excludes other CALD groups who do not speak the languages. Bilingual staff employed on trials may offer a beneficial alternative to interpreters and are able to build rapport with participants.

### Logistics

There are often practical and logistical issues to participation including transport, compensation, child care and time commitments [21, 24, 38, 43]. Travel to and from study sites can take time and cost money for participants, particularly those who reside in rural and regional areas and can be difficult to factor in on top of regular medical visits [23]. The impacts of participating in a trial should be addressed as much as possible. Studies and trials should be flexible, working to accommodate participants by adjusting appointment times, having trial sites in easy-to-access locations and community settings, and using alternative (remote) methods of data collection [2, 23, 38].

### Resources: funding and time

Actions to improve participation of CALD communities come with additional time, staffing and cost requirements. Research teams and organisations should be aware of these and ensure they are calculating their requirements accurately. As discussed above, toolkits and frameworks can be employed at the design phase to assist with this. The experts consulted acknowledge that these toolkits are often time-consuming, however, they stressed the importance of taking the time to complete this stage which will ensure that actions can be appropriately timed and budgeted for, creating a more sustainable study plan. Furthermore, the experts discussed the time and cost required for training staff, hiring community liaisons, recruitment in hospital settings requiring longer sessions, and building effective connections with CALD communities, a process that cannot be rushed and may take many conversations over a period of months, or longer.

it’s got to be a whole of system approach to some degree to address a big issue like this. And I think we need to encourage funders and ethics committees and that kind of thing to get on board with providing and incentivising researchers to be more inclusive… because otherwise there’s this great inertia and no real incentives for things to change.

Acquiring funding for these additional activities can be difficult with limited funds available, competitive grant application processes, and funding bodies often giving less than requested in applications by cutting community engagement. Requirements from sponsors and funding bodies to prioritise diversity in research as a condition of funding, alongside additional funding for these activities, would encourage diversification in research [1, 19].

## Conclusion

Improving the inclusion of CALD communities requires planning from the outset of a study to fully integrate actions throughout the trial period. Research teams must work directly with CALD communities to understand their unique needs and build sustainable and mutually beneficial connections, while understanding the resources required to achieve a truly inclusive research environment. While change is required from within research teams, top-down action from organisations, sponsors and funding bodies is required to help researchers create sustainable, long-term change for improved inclusion of CALD communities.

## Data Availability

The data generated during the current study are not publicly available as they contain details that could identify the experts consulted on this paper.

## Abbreviation

CALD: Culturally and linguistically diverse
